# The microRNA-17 ~ 92 Family as a Key Regulator of Neurogenesis and Potential Regenerative Therapeutics of Neurological Disorders

**DOI:** 10.1007/s12015-020-10050-5

**Published:** 2020-10-08

**Authors:** Xiaohuan Xia, Yi Wang, Jialin C. Zheng

**Affiliations:** 1 Center for Translational Neurodegeneration and Regenerative Therapy, Shanghai Tenth People’s Hospital affiliated to Tongji University School of Medicine, Shanghai, 200072 China; 2 Collaborative Innovation Center for Brain Science, Tongji University, Shanghai, 200092 China; 3 Departments of Pharmacology and Experimental Neuroscience, University of Nebraska Medical Center, Omaha, NE 68198-5930 USA

**Keywords:** Neural Stem/Progenitor Cells, microRNAs, Neurogenesis, Regenerative Medicine, miR-17~92, Proliferation, Differentiation

## Abstract

miR-17 ~ 92, an miRNA family containing three paralogous polycistronic clusters, was initially considered as an oncogene and was later demonstrated to trigger various physiological and pathological processes. Emerging evidence has implicated miR-17 ~ 92 family as a master regulator of neurogenesis. Through targeting numerous genes that affect cell cycle arrest, stemness deprivation, and lineage commitment, miR-17 ~ 92 family controls the proliferation and neuronal differentiation of neural stem/progenitor cells in both developmental and adult brains. Due to the essential roles of miR-17 ~ 92 family, its misexpression is widely associated with acute and chronic neurological disorders by attenuating neurogenesis and facilitating neuronal apoptosis. The promising neurogenic potential of miR-17 ~ 92 family also makes it a promising “medicine” to activate the endogenous and exogenous regenerative machinery, thus enhance tissue repair and function recovery after brain injury. In this review, we focus on the recent progress made toward understanding the involvement of miR-17 ~ 92 family in regulating both developmental and adult neurogenesis, and discuss the regenerative potential of miR-17 ~ 92 family in treating neurological disorders.

**Figure:**
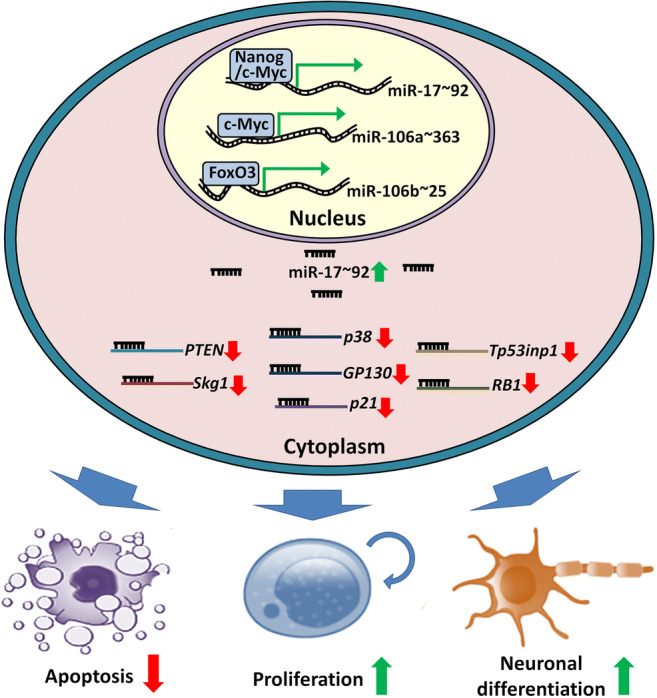
Graphical abstract

Graphical abstract

## Introduction

Neurogenesis is a fundamental process for both neural development and adult brain plasticity through which functional new neurons are generated from neural stem/progenitor cells (NSCs) [1, 2]. NSCs are a subset of undifferentiated precursors that are characterized by several features: (1) NSCs generate neural tissue or are derived from the nervous system; (2) NSCs retain the ability for proliferation and self-renewal; and (3) NSCs have the capacity to give rise to neuronal and glial lineages through asymmetric cell division [2]. Neurogenesis generally occurs throughout life in the subventricular zone (SVZ) of the lateral ventricles and the subgranular zone (SGZ) of the dentate gyrus, two distinct regions in mammalian brain [3]. The abnormality of neurogenesis is associated with the pathogenesis of various neurological disorders such as neurodegenerative diseases and schizophrenia [4]. For example, the impairment of adult neurogenesis that occurs in neurodegenerative diseases including Alzheimer’s disease (AD), Parkinson’s disease (PD), Huntington’s disease (HD), and amyotrophic lateral sclerosis (ALS) leads to the loss or concession of adult brain’s endogenous regenerative capacity and the putative function of newborn neurons, contributing to disease initiation and progression [4, 5].

The proper regulation of neurogenesis is essential for the development and function of the brain. The intracellular regulatory network of neurogenesis composed of microRNA (miRNA), transcription factors, epigenetic modification, and other factors, coordinates with extracellular cues to determine the spatial and temporal expression of essential genes that control the proliferation, fate specification, and differentiation of NSCs [6]. miRNAs are a class of highly conserved small noncoding antisense RNAs (20–24 nucleotides) that are originally discovered in Coenorhabditis elegans in 1984 [7]. miRNAs are transcribed from endogenous hairpin-shaped transcripts by RNA polymerase II or III [8]. Their transcripts, pri-miRNAs, are cleaved into pre-miRNAs in the nucleus by the Drosha/DGCR8 complex, and then exported to cytosol for another cleavage by the Dicer/TRBP complex [9]. The cleaved double stranded RNAs are separated to mature into miRNAs. After being expressed, miRNAs predominantly serve as a post-transcriptional silencer via either inducing the degradation of certain transcripts or interfering with translation process by binding to the 3’ untranslated region (UTR) of transcripts [10]. miRNAs have emerged as a crucial regulator in both development and adult neurogenesis [7, 11–14]. The cortex-specific knockout (KO) of Dicer, a key enzyme for miRNA biogenesis, significantly reduces the cellular complexity during cerebral cortex development [15]. *In vitro* studies also demonstrated that the deletion of Dicer blocks the differentiation of embryonic NSCs into highly diverse types of neurons [16]. Instead, only one class of deep layer projection neurons is continually produced, suggesting the loss of multipotency and neuronal lineage progression in miRNA-depleted NSCs. Similarly, the removal of most miRNAs in retina by Dicer KO leads to massive death of retinal progenitor cells and neurogenesis deficiency in both embryonic and neonatal stages [17]. Moreover, Dicer ablation impairs neurogenesis, but not astrogliogenesis, in the adult hippocampus, which, is confirmed in an *in vitro* adult neurogenesis model [18]. These observations suggest the key function of miRNAs in the maintenance of NSC pool, the lineage commitment of NSCs, and the maturation of differentiated NSCs in both developmental and adult brains. The abnormal expression of miRNAs is greatly associated with various neurological disorders including acute brain injury and chronic neurodegenerative diseases [19]. Therefore, miRNAs are broadly investigated as novel drug targets and biomarkers of neurological disorders.

To date, multiple miRNA clusters have been discovered and, among them, microRNA-17 ~ 92 (miR-17 ~ 92) family has been considered as one of the most important stem cell regulators [20, 21]. miR-17 ~ 92 polycistron was firstly identified as an oncogene due to its abnormally elevated expression levels in and pro-proliferative effects on multiple types of tumor cells, such as diffuse large B-cell lymphomas, mantle cell lymphomas, and Burkitt’s lymphomas cells [22, 23]. Afterwards, the expression of miR-17 ~ 92 family has been detected in many organs including the brain, especially in developmental phases [20, 24, 25]. Merging evidence has implicated miR-17 ~ 92 family in regulating neurogenesis via facilitating NSC proliferation, suppressing NSC differentiation, and inhibiting apoptosis [20, 26–31]. miR-17 ~ 92 family achieves its function through targeting various anti-neural or anti-proliferative genes including *PTEN*, *Tp53inp1*, and *p21* [26, 32]. Due to its importance in neurogenesis regulation, miR-17 ~ 92 family is widely involved in the pathogenesis of neurobiological disorders. For instance, the decay and over-synthesis of miR-17 ~ 92 are linked to neurogenesis deficiency in neurodegenerative diseases and uncontrolled cell growth in glioma, respectively [33, 34]. Conversely, miR-17 ~ 92 family can also be utilized as potential regenerative therapeutics to treat brain injury via stimulating endogenous neurogenesis [35]. This review will summarize recent progress made toward understanding the involvement of miR-17 ~ 92 family in regulating both developmental and adult neurogenesis, describe the pathological effects of miR-17 ~ 92 in neurological disorders, and provide a discussion of the regenerative capacity of miR-17 ~ 92 family in treating neurological disorders.

## miR-17 ~ 92 Family: Members and Classification

**Fig. 1:**
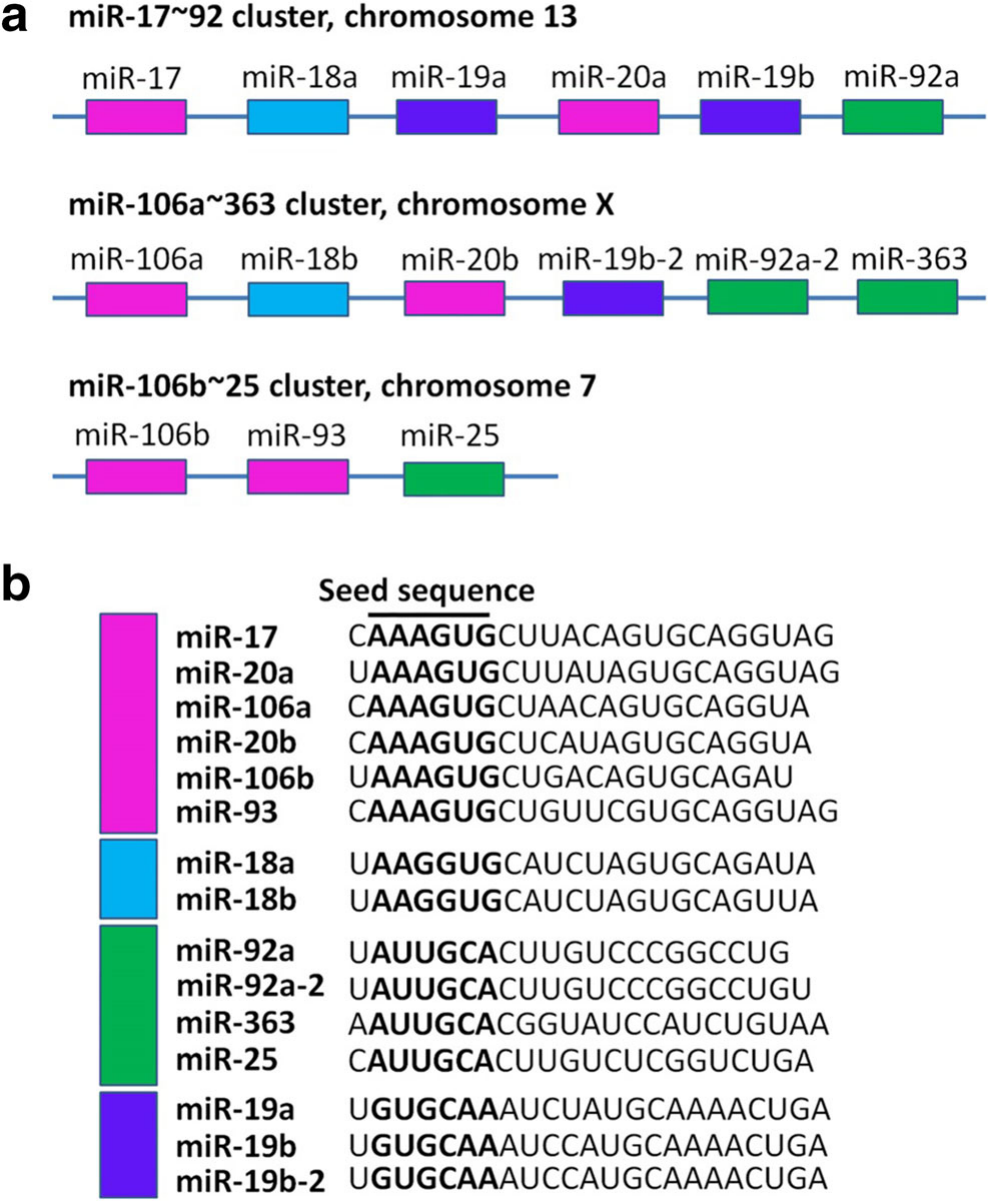
Gene structure of human miR-17 ~ 92 family. **A** Transcript organization of the human miR-17 ~ 92 family, including miR-17 ~ 92 cluster and its paralogs, miR-106a ~ 363 and miR-106b ~ 25 clusters. miR-17 ~ 92 cluster locates in chromosome 13 and comprises six miRNAs. miR-106a ~ 363 cluster locates in chromosome X and comprises six miRNAs as well. miR-106b ~ 25 cluster locates in chromosome 7 and comprises three miRNAs. Each cluster is transcribed as a single transcript, but differentially processed thereafter. **B** miR-17 ~ 92 family miRNAs are grouped into four sub-families including miR-17/106 (miR-17, miR-20a, miR-20b, miR-106a, miR-106b and miR-93), miR-18 (miR-18a and miR-18b), miR-19 (miR-19a, miR-19b-1, and miR-19b-2), and miR-25/92 (miR-92a-1, miR-92a-2, miR-383, and miR-25) sub-families, according to their seed sequences. Seed sequences are shown in bold. miR/miRNA: microRNA

miR-17 ~ 92 family consists of three paralogous polycistronic clusters: miR-17 ~ 92 cluster (miR-17, miR-18a, miR-19a, miR-20a, miR-19b-1, and miR-92a-1), miR-106b ~ 25 cluster (miR-106b, miR-93, and miR-25), and miR-106a ~ 363 cluster (miR-106a, miR-18b, miR-20b, miR-19b-2, miR-92a-2, and miR-363) (Fig. 1A) [20]. These clusters are with high similarity and identical 7mer seed sequences, but with different chromosomal locations. miR-17 ~ 92 cluster is located in the 13q31.3 region of human chromosome 13, tightly grouped within an 800 base-pair region, and transcribed as a single polycistronic unit [36]. Otherwise, miR-106b ~ 25 and miR-106a ~ 363 clusters are located on human chromosome 7 and the X chromosome, respectively [25]. Besides, miR-17 ~ 92 family can also be classified by their seed sequence (nucleotides 2–8), since miRNAs recognize their targets via the binding of seed sequence with complementary sequences on mRNA. In this way, miR-17 ~ 92 family can be clustered into four sub-families, miR-17/106 sub-family (miR-17, miR-20a/miR-20b, miR-106a/miR-106b, and miR-93), miR-18 sub-family (miR-18a/miR-18b), miR-19 sub-family (miR-19a/miR-19b), and miR-25/92 sub-family (miR-25, miR-92a, and miR-363) (Fig. 1B) [25]. The unique expression machinery and distinct seed sequence of miR-17 ~ 92 family members suggest that these miRNAs play in concert in the regulation of certain biological processes including neurogenesis. To understand the exact role of miR-17 ~ 92 family in neurogenesis, multiple approaches that modulate the expression of entire miRNA cluster and specific miRNA in the cluster have been carried out, and the complex network of miR-17 ~ 92 family in the regulation of NSCs has been gradually unveiled.

## miR-17 ~ 92 Family in Developmental Neurogenesis

The involvement of miR-17 ~ 92 family in developmental neurogenesis is firstly prompted by multiple temporal expression analyses *in vivo* and *in vitro*. In 2003, Krichevsky et al. identified that miR-17 ~ 92 family is highly expressed in embryonic mouse brain but not in adult one through a miRNA array [37]. Similarly, Mao et al. reported that the expression levels of miR-17 ~ 92 family miRNAs gradually reduced with cortex development [38]. More importantly, the change in miR-17 expression occurred in the ventricular zone/sub-ventricular zone, where NSCs located. The downward trend of miR-17 ~ 92 family expression levels during mouse embryonic NSC differentiation is confirmed by miRNA array in an *in vitro* model of developmental neurogenesis, implying that miR-17 ~ 92 family is strongly associated with NSC regulation during brain development [26].

This premise is firstly proved by generating Emx1^+^ cortical cell-specific miR-17 ~ 92 single-KO mice [28]. The KO mice exhibit reduced cortical thickness, fewer NSCs in embryonic cortex, enhanced transition from NSCs to intermediate progenitors (IPs), and dysregulated generation of neurons during brain development. Similar results were observed when either miR-106a ~ 363 or miR-106b ~ 25 was deleted together with miR-17 ~ 92, suggesting that miR-17 ~ 92 family share homologous function in regulating embryonic NSCs. After that, the roles of individual miR-17 ~ 92 family miRNA in the regulation of NSCs were examined. The ectopic expression of miR-17 or miR-106b, two miR-17/106 sub-family miRNAs, enhances the proliferation of embryonic cortical NSCs, therefore maintains the NSC pool in the developing cerebral cortex [26, 38, 39]. The knockdown of miR-17 and miR-20, by contrast, significantly inhibits the proliferation of mouse cerebella NSCs, ascertained by EdU incorporation assay, neurosphere counting, and FACS-based cell cycle analysis [27]. Another key miRNA in miR-17 ~ 92 family, miR-92, also participates in maintaining NSC self-renewal in developing cortex, suggesting the involvement of miR-25/92 sub-family in the proliferation of NSCs [40]. Except for the pro-proliferative capacity, miR-17 ~ 92 family is also involved in the commitment of different lineages during NSCs differentiation [31]. The ectopic expression of either miR-17 or miR-106b significantly increases the proportions of neurons and decreases that of astrocytes in an *in vitro* neurogenesis model and in the developing mouse forebrains, suggesting miR-17/106 sub-family serves as one key roadblock for the neurogenic-to-gliogenic transition [31]. Additionally, miR-18 sub-family plays an important role in neuronal differentiation in brain development as well. The morpholino-induced knockdown of miR-18a and miR-18b accelerates the generation of mature cone photoreceptor, a specified group of neurons that are differentiated during early retinal histogenesis, in zebrafish [41].

Taken together, mounting evidence has implicated miR-17 ~ 92 family as a key regulator in developing brain. However, the thorough roles of this family in this process are far away from being fully understood. Although all miR-17 ~ 92 family miRNAs exhibit similar expression profiles during brain development, their function may vary due to distinct seed sequence. Currently, the function of miR-17/106 sub-family in neurogenesis is under extensive investigation. In contrast, the function of miR-19 or miR-25/92 sub-family is rarely studied. Thus, to explore the involvement of each sub-family is important to fill the aforementioned knowledge gap. Surprisingly, even though the same seed sequence is shared, each member of miR-17/106 sub-family displays unequal efficiency in NSC regulation [26]. Parallel comparison demonstrated that miR-106b has higher capacities in facilitating the proliferation of NSCs and suppressing neurogenic-to-gliogenic transition than miR-106a, suggesting that the effects of miR-17 ~ 92 family miRNAs and their underlying mechanisms are much more complex than what we previously thought [31].

## miR-17 ~ 92 Family in Adult Neurogenesis

Neurogenesis in the brain of adult mammals occurs throughout life under both basal conditions and in response to injury [1, 3, 42]. In adult brain, neurogenesis starts from the generation of rapidly proliferating IPs from NSCs in the SVZ and SGZ. IPs ultimately differentiate into neurons, migrate into the olfactory bulb or the dentate gyrus, and mature into inhibitory interneurons or excitatory neurons to support brain function. High-throughput analysis identified miR-17 ~ 92 family miRNAs were down-regulated in cells isolated from old donors, compared with young ones [43]. In neurological disorders that display neurogenesis disruption like Down syndrome and schizophrenia, the expression levels of miR-17 ~ 92 family members and their host gene are also observed to be repressed in adult NSCs [29, 44, 45]. Moreover, studies have shown decreased expression of miR-17 ~ 92 family miRNAs during adult NSCs differentiation, suggesting a link between miR-17 ~ 92 family and adult neurogenesis [46].

The roles of miR-17 ~ 92 family in adult neurogenesis are examined by perturbation of function approaches. miR-17 ~ 92 family depletion suppresses neurogenesis, while its overexpression enhances neurogenesis, likely through regulating the expansion of NSC pool in the dentate gyrus of adult mice [47, 48]. Similar results were observed that the ectopic expression of miR-106b ~ 25 cluster leads to an increase in the proliferation and neuron production capacities of adult NSCs [30]. miR-25 was identified as the main effector, since the manipulation of expression levels of either miR-93 or miR-106b did not affect NSC proliferation. Under ischemic conditions, miR-25 positively regulates adult NSC proliferation in the SVZ, providing evidence for miR-17 ~ 92 family-mediated NSC proliferation *in vivo* [49]. In addition, miR-106b ~ 25 cluster also regulates the cell fate commitment of adult NSCs [30]. The overexpression of miR-17 ~ 92 family in general and miR-106b ~ 25 cluster in particular both promote the generation of neurons from NSCs, suggesting miR-17 ~ 92 family can shift the differentiation preference of NSCs bias towards neuronal lineage [47]. Besides, miR-17 ~ 92 family controls the migration ability of adult NSCs. In an *in vitro* cell migration assay, migrated newborn neurons express more miR-19 than unmigrated ones [46]. Perturbation of function assay then demonstrated that the migration efficiency of adult NSCs increased when miR-19 was overexpressed and decreased when miR-19 was knocked down. These studies provide a new perspective in viewing miR-17 ~ 92 family’s contribution in neurogenesis other than NSC fate commitment.

It is worth-noting that our knowledge on the effects of miR-17 ~ 92 family on adult NSCs is incomplete due to the lack of comprehensive investigations of individual miRNAs in this family. Compared with studies on developing brains, fewer groups concentrate on the involvement of miR-17 ~ 92 family in adult neurogenesis largely due to the doctrinal debate of the existence of adult NSCs in human brain, the higher technical threshold of adult NSC isolation and gene perturbation, and the fuzzier role of unconspicuous neurogenesis in normal adult brain. Based on current literatures, interesting results have been reported. Unlike the situation in developing brain, miR-25/92 sub-family may be more important than miR-17/106 in regulating the proliferation and differentiation of adult NSCs [30]. However, only miR-106b is used to compare with miR-25, and more miRNAs in miR-17/106 sub-family should be examined to confirm this finding. Thus, although studies demonstrate miR-17 ~ 92 family as an indispensable regulator of adult neurogenesis, our knowledge remains limited and more detailed research is urgently needed.

## The Regulatory Networks of miR-17 ~ 92 Family

### The Down-stream Targets of miR-17 ~ 92 Family

The aforementioned discrepancy of miR-17 ~ 92 family miRNAs in the context of NSC regulation indicates that each sub-family exhibits distinct functions by targeting different genes. Therefore, to identify the targets of miR-17 ~ 92 family miRNAs is an essential task.

**Fig. 2:**
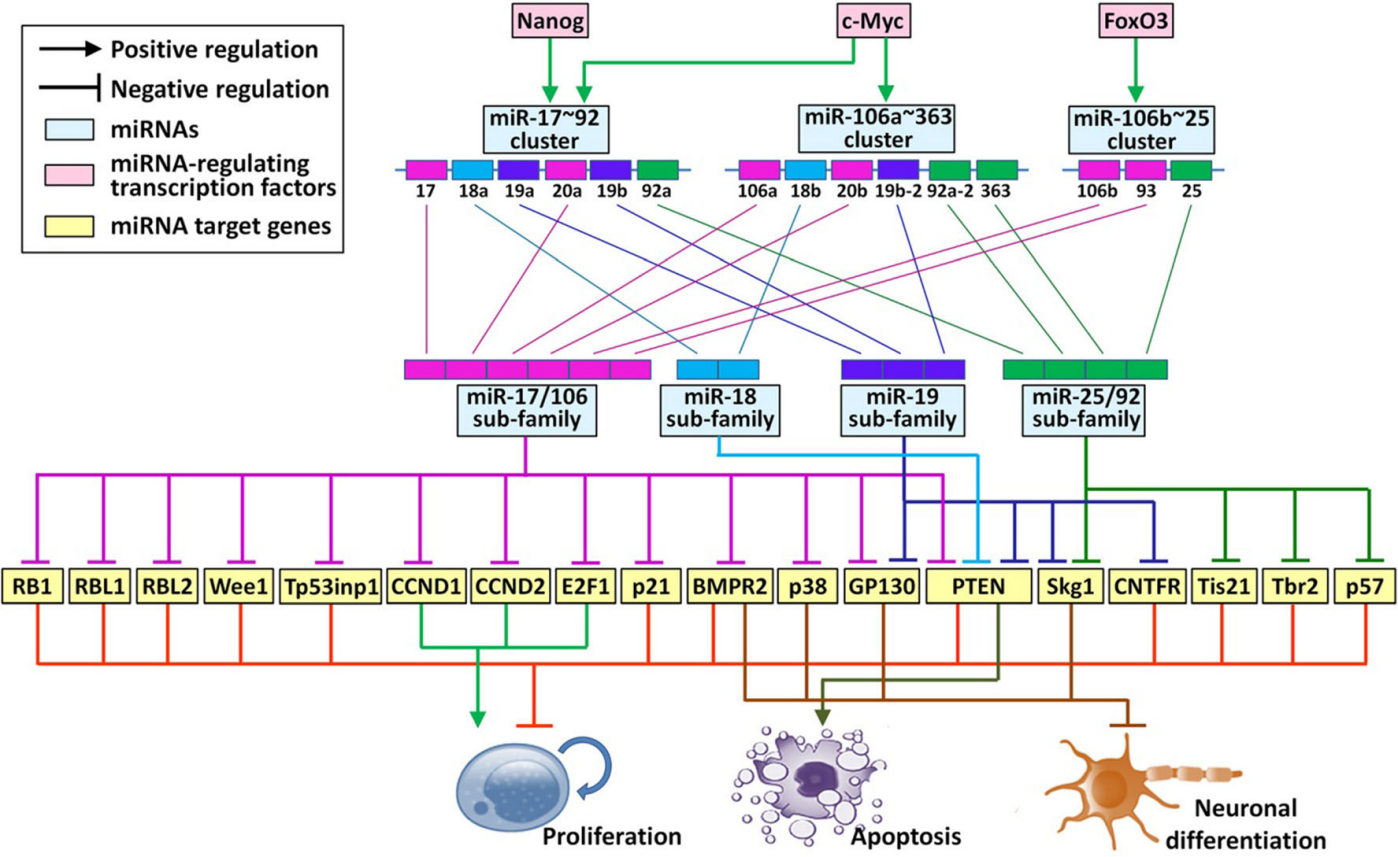
Mechanisms of miR-17 ~ 92 family on the regulation of neurogenesis. The expression of miR-17 ~ 92 family in NSCs is regulated by multiple transcription factors including c-Myc, Nanog, and FoxO3. After being expressed, miR-17 ~ 92 family miRNAs inhibit the expression of their target genes via the direct binding of miRNA seed sequence to the 3’ UTR of transcripts, leading to the enhancement of proliferation, the acceleration of neuronal differentiation, and the suppression of apoptosis. Therefore, miR-17 ~ 92 family-related regulatory networks function as a key controller of the developmental and adult neurogenesis. miR/miRNA: microRNA, UTR: untranslated region

To date, numbers of targets have been identified for miR-17/106 sub-family (Table 1; Fig. 2). Garg et al. and our group both observed that miR-17/106 sub-family miRNAs (miR-17, miR-20, and miR-106) target key components of p53 signaling, Trp53inp1 and p21 [26, 27, 39]. TSG101-Trp53inp1-p53-p21 is a key axis in modulating cell growth arrest and apoptosis [50]. The inhibition of *Trp53inp1* and *p21* expression by miR-17/106 sub-family attenuates p53 signaling, supporting the self-renewal of NSCs and preventing the premature exhaustion of NSC pool. miR-17/106 sub-family has also been reported to target *p38*, *BMPR2*, *GP130*, *RB1*, *RBL1*, *RBL2*, *Wee1*, *CCND1*, *CCND2*, *E2F1*, and *PTEN*, therefore repressing gliogenic MAPK and BMP2 pathways [31, 32, 38, 51].

**Table 1:** Validated targets of miR-17 ~ 92 family in neurogenesis

miR-17 ~ 92 sub-families	Target genes	Affected pathways	Target’s effects	Reference
miR-17/106 sub-family	*p38*	MAPK pathway	Gliogenesis initiation	[31]
	*PTEN*	PI3K-Akt & Fas/FasL pathways	Anti-proliferation, pro-apoptosis	[32]
	*GP130*	JAK-STAT pathway	Neuronal differentiation inhibition	[51]
	*BMPR2*	BMP pathway	Proliferation suppression, neuro-to-gliogenesis transition	[38]
	*p21*	Cell cycle-related pathway	Cell cycle arrest	[26, 32]
	*Tp53inp1*	p53 signaling	Anti-proliferation	[26]
	*RBL1/2*	p53 signaling	Anti-proliferation	[32]
	*RB1*	Notch pathway	Anti-proliferation	[32]
	*Wee1*	Cell cycle-related pathway	Cell cycle arrest	[32]
	*CCND1/2*	Cell cycle-related pathway	Pro-proliferation	[32]
	*E2F1*	p38-HSP27 pathway	Pro-proliferation	[32]
miR-18 sub-family	*PTEN*	PI3K-Akt & Fas/FasL pathways	Proliferation inhibition, apoptosis activation	[52]
miR-19 sub-family	*Sgk1*	Glucocorticoid pathway	Hippocampal proliferation inhibition	[48]
	*PTEN*	PI3K-Akt & Fas/FasL pathways	Proliferation inhibition, apoptosis activation	[52]
	*GP130*	JAK-STAT pathway	Neuronal differentiation inhibition	[51]
	*CNTFR*	JAK-STAT pathway	Neuronal differentiation inhibition	[51]
miR-25/92 sub-family	*Sgk1*	Glucocorticoid pathway	Hippocampal proliferation inhibition	[48]
	*p57*	Cell cycle-related pathway	Cell cycle arrest	[49]
	*Tis21*	BMP & Notch pathways	Anti-proliferation, pro-differentiation	[40]
	*Tbr2*	Histone demethylation-related pathway	Anti-proliferation, neuronal differentiation promotion	[28]

Mounting studies also report the direct targeting of miR-25/92 sub-family with multiple regulators of NSCs. miR-92 binds to *Tbr2* and *Tis21* to down-regulate their expression, leading to the repression of IPs expansion and the maintenance of NSC pool in developing cortex [28, 40]. miR-92 also binds to *Skg1* to regulate glucocorticoid pathway in NSCs and rescue hippocampal proliferation caused by corticosterone [48]. miR-25 is reported to repress cell cycle arrest through targeting *p57* under ischemic conditions, thus promoting the proliferation of adult NSCs in the SVZ [49]. The bioinformatics of functional annotation further identified multiple miR-25 target mRNAs belonging to insulin/insulin-like growth factor-1 (IGF) signaling [30]. Besides, studies on tumor cells also reported that miR-25 targets *REST*, a key anti-neural transcription silencer, implying another potential mechanism for miR-25-mediated NSC regulation [53]. Although the binding of miR-25 to its predicted targets and the functions of miR-25 targets require further validation, this result suggests that miR-25/92 may regulate neurogenesis through multiple pathways.

Due to the lack of studies, the information for the targets of either miR-19 or miR-18 sub-family in NSCs remains limited. Currently, only a few targets of miR-19 sub-family are confirmed, including *PTEN*, *Skg1*, *GP130*, and *CNTFR* [28, 48, 51]. Based on the studies of cancer cells or stem cells out of the central nervous system (CNS), miR-19 facilitates proliferation via targeting *HIPK1* [54], *Plzf* [55], and *Cyld* [56]. Besides, miR-18 regulates tumorigenesis by suppressing *SOCS5* [57], *CTGF*, *Nedd9*, *IGF1*, and *CDK19* [58]. However, whether or not these genes are also expressed in NSCs and regulated by miR-18/19 sub-families need to be further clarified.

To date, dozens of miR-17 ~ 92 family targets have been identified. However, being a key post-transcriptional regulator, miR-17 ~ 92 family have thousands of predicted targets. Thus, only a very small proportion of targets have been confirmed, especially in the field of NSC regulation. Under this circumstance, detailed screening is required to identify and confirm the down-stream axis in miR-17 ~ 92-regulated neurogenesis.

### The Up-stream Regulators of miR-17 ~ 92 Family

The decline of expression levels of miR-17 ~ 92 family miRNAs during neurogenesis suggests that this family is under precise and integrated regulation. Nanog is the first transcription factor that is reported to bind to the up-stream regulatory region of miR-17 ~ 92 family and maintain high levels of transcription of the latter (Fig. 2) [27]. After that, FoxO3, an insulin/IGF signaling-down-stream transcription factor is found to target the first intron of miR-106b ~ 25 cluster, implying that insulin/IGF signaling may promote miR-25 expression in a feedback manner to maintain adult NSCs and extend cell lifespan [30]. In addition, the promoter of miR-17 ~ 92 family can also be occupied by other transcription factors including c-Myc, E2F1, and C/EBP-β in tumor cells [59, 60]. Although the direct binding of c-Myc with miR-17 ~ 92 encoding gene in NSCs remains unproven, the up-regulation of c-Myc raises the expression levels of miR-17 ~ 92 family miRNAs, therefore modulating neurogenesis [52]. Moreover, both E2F1 and C/EBP-β are expressed in NSCs and their expression levels decrease over the course of neuronal differentiation, positively correlated with the expression patterns of miR-17 ~ 92 family [61, 62]. This correlation implies an association of E2F1 and C/EBP-β with miR-17 ~ 92 family miRNA expression in NSCs, although the exact roles of E2F1 and C/EBP-β are to be proved.

Taken together, multiple down-stream and up-stream factors of miR-17 ~ 92 family have been identified in NSCs, which establish a complicated and precise regulatory network in controlling the maintenance and fate commitment of NSCs.

## miR-17 ~ 92 Family as Pathological Factor in Neurological Disorders

Due to the key roles of miR-17 ~ 92 family in NSC regulation, its abnormal expression is tightly associated with various neurological disorders (Table 2).

**Table 2:** The pathological effects of miR-17 ~ 92 family on neurological disorders

Neurological disorders	Expression trends of miR-17 ~ 92	Effects of miR-17 ~ 92	Targets	Reference
Stroke	Down-regulation of miR-17	Cell death resistance	*PTEN*, PI3K/Akt/mTOR pathway	[35]
	Down-regulation of miR-17	Anti-apoptosis	Fas/FasL pathway	[63]
Alzheimer’s disease	Down-regulation of miR-17, -20a, and -106	Aβ plaque and Tau phosphorylation inhibition	N/A	[64, 65]
	Up-regulation of miR-25	Anti-proliferation, pro-apoptosis	*KLF2*	[66]
Parkinson’s disease	Down-regulation of miR-17	Pro-proliferation and dopaminergic neuron survival	N/A	[33]
Glioma	Up-regulation of miR-17 ~ 92	Glioma cell growth, anti-apoptosis	*p21*, *E2F1*, *PTEN*	[67]
	Up-regulation of miR-19	Drug resistance enhancement	*MDR-1*	[68–70]

In an *in vitro* cerebral hypoxia/reperfusion (H/R) model, miR-17 expression is inhibited in hypoxia-exposed human brain microvascular endothelial cells [71]. The expression of pro-apoptotic genes such as *PTEN* and the activities of PI3K/AKT/mTOR signaling were elevated without the presence of miR-17, causing severe cell death. In another H/R model that is estabolised by oxygen-glucose deprivation (OGD) also showed significant down-regulation of miR-25 [63]. The reduction of miR-25 expression released Fas/FasL pathway from inhibition, leading to cell apoptosis. These findings suggest great contribution of miR-17 ~ 92 family deregulation to the initiation and progression of acute brain injury.

In neurodegenerative diseases that exhibit significant impairment of endogenous neurogenesis, the expression of multiple members of miR-17 ~ 92 family is severely compromised, which is involved in disease pathogenesis. For instance, miR-17, -20a, and -106 have consistently shown deregulation in the cortical and hippocampal tissues in experimental models and human samples of AD [64, 65]. The decay of miR-17 ~ 92 family can be associated with the production of Aβ plaque and hyperphosporylation of Tau, although the exact mechanisms remain ambiguous [64, 65]. However, Duan and Si reported an unexpected elevation of miR-25 expression levels in hippocampal tissues of AD mice [66]. The misexpression of miR-25 can inhibit proliferation and induce cell apoptosis in AD mouse primary hippocampal cell culture, suggesting miR-17 ~ 92 may possess much more complex functions in the pathogenesis of neurodegenerative diseases than previously thought. Furthermore, in an *in vitro* PD model, the expression levels of miR-17 are significantly reduced, contributing to dopaminergic neurodegeneration and proliferation attenuation [33].

Besides, being a well-recognized primary oncogenic miRNA family, the expression of miR-17 ~ 92 is dramatically up-regulated in glioma tissues. miR-17 ~ 92 family facilitates the uncontrolled growth of glioma cells [34], and inhibition of miR-17 ~ 92 family decreases proliferation and induces apoptosis of glioblastoma cells by elevating the expression levels of *p21*, *E2F1*, and *PTEN* [67]. In addition, miR-17 ~ 92 family, especially miR-19, plays an important role in drug resistance via regulating multidrug resistance (MDR)-related transporters including MDR-1 [68–70].

## miR-17 ~ 92 Family as Regenerative “Medicine”

Due to its crucial roles in enhancing proliferation, neuronal fate commitment, and migration of NSCs in both physiological [26] and pathological conditions [49], miR-17 ~ 92 family has been applied as potential regenerative “medicine” for treating acute CNS disorders (Table 3). For example, miR-17 ~ 92 family miRNAs are specifically loaded into mesenchymal stromal cells (MSCs)-derived exosomes for enhancing neuroplasticity and functional recovery after stroke in rats [35]. miR-17 ~ 92 family-enriched exosomes display significantly more robust effects on improving neurological function and enhancing oligodendrogenesis, neurogenesis, and neurite remodeling/neuronal dendrite plasticity, compared with control MSC exosomes or liposomes, after being intravenously administrated into the brain of middle cerebral artery occlusion (MCAO) rat. Follow-up studies demonstrated that miR-17 ~ 92 family achieves regenerative capacity likely due to the inhibition of PTEN-mTOR-GSK3β pathway.

**Table 3:** The therapeutic effects of miR-17 ~ 92 family on neurological disorders

Neurological disorders	Administration route	Effects of miR-17 ~ 92	Targets	Reference
Stroke	Intravenous administration of miR-17-enriched MSC exosomes	Neuroplasticity and functional recovery enhancement	*PTEN*, PI3K/Akt/mTOR pathway	[35]
Hypoxic-ischemic encephalopathy	Intracerebroventricular infusion of miR-17 mimics	Inactivation of NLRP3 inflammasome, anti-apoptosis	*TXNIP*	[72, 73]
Traumatic brain injury	Ectopic expression of miR-17 ~ 92 cluster in grafted NSCs	Atrocytogenesis inhibition, neurogenesis promotion	*LIF*/*CNTF*	[51]

miR-17 ~ 92 family is utilized to promote the neurogenic potential of exogenous NSCs post brain transplantation under a traumatic brain injury (TBI)-induced neuroinflammatory conditions as well [51]. By overexpressing miR-17 ~ 92 family, transplanted NSCs exhibit increased neurogenesis and reduced astrogliosis. More importantly, miR-17 ~ 92-overexpressed NSC transplantation significantly improves the motor coordination of TBI mice, versus control NSC treatment, proposing that miR-17 ~ 92 as a promising “medicine” to accelerate neurogenesis and functional recovery after brain injury.

The enhancement of miR-17 ~ 92 family expression also exerts neuroprotective effects, other than promoting neurogenesis. For example, miR-17 ~ 92 family can inhibit neuronal apoptosis and neuroinflammation in neonatal hypoxia-ischemia (HI) rats, a model of hypoxic-ischemic encephalopathy [72, 73]. miR-17-mediated neuroprotection is highly likely achieved by the suppression of TNXIP-induced activation of ASK1/p38 pathway and NLRP3 inflammasome.

Although promising results have been obtained for the use of miR-17 ~ 92 family in treating acute brain injury, there is still a long way to utilize miR-17 ~ 92 from bench to bed side. Similar to other *in vivo* RNA molecule delivery attempts, the main challenges for the clinical application of miR-17 ~ 92 include enhancing bioavailability, achieving targeted delivery, prolonging half-life *in vivo*, and reducing side effects. Naked RNA molecules get rapidly degraded *in vivo*, are accumulated in organs like kidney, fail to diffuse across the blood-brain barrier (BBB), and exhibit no targeting potential. Thus, multiple synthetic or natural nanocarriers have been applied in miR-17 ~ 92 delivery, including aforementioned exosomes. Emerging evidence has implicated exosomes as an excellent natural platform for delivering miRNA into the CNS due to superior miRNAs preservation from the RNases, promising targeting capacity post equipping with homing molecules, ability to penetrate the BBB, low- or non-immunogenicity, and flexibility in administration routes [74–78]. These unparalleled characteristics make exosome-based miRNA delivery an interesting and important direction for the development of novel therapeutic strategies for treating neurological disorders.

It is also important to emphasize that there remains an uncharted territory for investigating the therapeutic roles of miR-17 ~ 92 family in neurodegenerative diseases *in vivo*. Featured by neurogenesis deficiency, to successfully treat neurodegenerative diseases requires restored neuronal generation to replace degenerating neurons. Currently, pioneer studies have demonstrated the potential therapeutic effects of miR-17 ~ 92 family in various neurodegenerative diseases *in vitro*. For instance, miR-17 and miR-20a can reverse neurogenesis attenuation in an *in vitro* PD model and inhibit T cell activation genes in an *in vitro* Multiple Sclerosis model [79]. With more comprehensive research efforts, the application potential of miR-17 ~ 92 family in chronic neurodegenerative diseases may be unveiled in a near future.

## Conclusions and Future Perspectives

In this review, we have summarized current knowledge for the involvement of miR-17 ~ 92 family in the regulation of neurogenesis. The perturbation of function approaches implicate miR-17 ~ 92 family as master regulators of proliferation and neuronal differentiation in both developmental and adult brains via targeting numerous genes controlling cell cycle arrest, stemness deprivation, and lineage commitment. Inspiringly, pilot studies have been carried out to validate the potential of miR-17 ~ 92 family in treating neurological diseases by taking advantages of the regenerative capacity of these miRNAs, and positive results have been reported.

It is worth-noting that, except for neurogenesis regulation, miR-17 ~ 92 family is also associated with other physiological and pathological processes in the brain. For example, miR-17 ~ 92 family has been shown to repress neuronal apoptosis, NLRP3 inflammasome-mediated neuroinflammation, and tau phosphorylation in rodent models of stroke and AD, implying miR-17 ~ 92 family has multiple potential therapeutic effects not confined to activating neurogenesis [72, 73, 80]. Therefore, the systematic investigation of miR-17 ~ 92 family is an attractive and important direction to unveil the neurological functions of this family, shedding light on the development of novel therapeutic strategies for treating CNS disorders.

## Data Availability

Not applicable.

## Abbreviations

AD: Alzheimer’s disease
AIS: Acute ischemic stroke
ALS: Amyotrophic lateral sclerosis
BBB: Blood-brain barrier
CNS: Central nervous system
CSF: Cerebrospinal fluid
HD: Huntington’s disease
HI: Hypoxia-ischemia
H/R: Hypoxia/reperfusion
IGF: Insulin-like growth factor
IPs: Intermediate progenitors
KO: Knockout
MCAO: Middle cerebral artery occlusion
MDR: Multidrug resistance
miR-17 ~ 92: MicroRNA-17 ~ 92
miRNA: MicroRNA
MSCs: Mesenchymal stromal cells
NSCs: Neural stem/progenitor cells
OGD: Oxygen-glucose deprivation
PD: Parkinson’s disease
SGZ: Subgranular zone
SVZ: Subventricular zone
TBI: Traumatic brain injury
UTR: Untranslated region
